# BCR-JAK2 fusion as a result of a translocation (9;22)(p24;q11.2) in a patient with CML-like myeloproliferative disease

**DOI:** 10.1186/1755-8166-5-23

**Published:** 2012-05-01

**Authors:** Mohamed M Elnaggar, Sally Agersborg, Trilochan Sahoo, Ati Girgin, Wanlong Ma, Ronjay Rakkhit, Isabel Zorrilla, Alexis Leal

**Affiliations:** 1 Cytogenetics, Quest Diagnostics Nichols Institute, 33608 Ortega Highway, San Juan Capistrano, CA 92675, USA; 2 Memorial Hermann Memorial City Medical Center, Houston, TX 77024, USA

**Keywords:** CML, *JAK2*, *BCR*, Translocation, t(9;22)

## Abstract

Translocation (9;22)(q34;q11.2) resulting in BCR/ABL1 fusion at the molecular level is the hallmark of chronic myelogenous leukemia (CML). Variants of the Philadelphia translocation and complex translocations involving *BCR* have been reported in myeloproliferative disorders (MPD). A rare translocation, t(9;22)(p24;q11.2), resulting in a novel BCR-JAK2 fusion has been reported in a handful of cases of CML and acute myelogenous leukemia (AML). We present clinical-pathological and cytogenetic evaluation of a patient with Philadelphia-chromosome negative CML/MPD harboring a t(9;22)(p24;q11.2) resulting in BCR-JAK2 fusion. Fluorescence in situ hybridization and molecular characterization of the translocation confirmed a BCR-JAK2 fusion and helped delineate the breakpoints upstream of exon 1 of minor cluster region of *BCR* gene and likely intron 18 of the *JAK2* gene, resulting in an in-frame transcript This case provides convincing support, along with two previous case-reports, for a role for activation of the Janus kinase 2 in evolution of myeloproliferative disease. The recurrent, albeit rare, nature of the breakpoints within *BCR* and *JAK2* suggests a potential new diagnostic target that should be interrogated in Ph-negative CML/MPD patients.

## Introduction

The Philadelphia translocation is one of the most well characterized cytogenetic aberrations seen in a vast majority of cases of chronic myelogenous leukemia. The resulting oncogenic BCR-ABL1 fusion transcript retains tyrosine kinase activity and is the target of therapeutic tyrosine kinase inhibitors. Janus kinases (JAKs) are a family of receptor-associated tyrosine kinases that function through interaction with specific cytokine receptors, principally via signal transducers and activators of transcription (STATs) [1]. Janus kinase 2 gene (*JAK2*), a specific mediator of erythropoietin signaling, has been implicated in a whole variety of myeloproliferative neoplasms [2]‐[5]. A recurrent dominant gain-of-function mutation in *JAK2*, JAK2V617F, results in constitutional activation of its kinase domain and has been widely established to be causally related to chronic myeloproliferative disorders, particularly polycythemia vera. The somatic V617F gain-of- function mutation in exon 14 of *JAK2* gene, and less commonly exon 12 mutation of *JAK2* have found in greater than 95% of patients with polycythemia vera and about 50% of patients with essential thrombocythemia and myelofibrosis [6]‐[8]. Additionally, a single case report implicates a role for the V617F mutation of *JAK2* in de novo AML [9]. Interestingly, *JAK2* has been identified to be involved in two rare translocations: with *ETV6*, at 12p13, in acute lymphoblastic leukemia and rarely myeloproliferative (CML-like) disorder [4,10] and with *BCR*, at 22q11.2, in patients with chronic myeloid leukemia [11]. Here we report a case of chronic myeloid leukemia (CML) with a translocation (9;22)(p24;q11.2), resulting in BCR-JAK2 fusion, as a sole cytogenetic abnormality. The fusion gene was confirmed at the molecular level. This case report provides additional strong support for a role for *JAK2* activation in chronic myeloproliferative disorders.

## Clinical report

**Figure 1:**
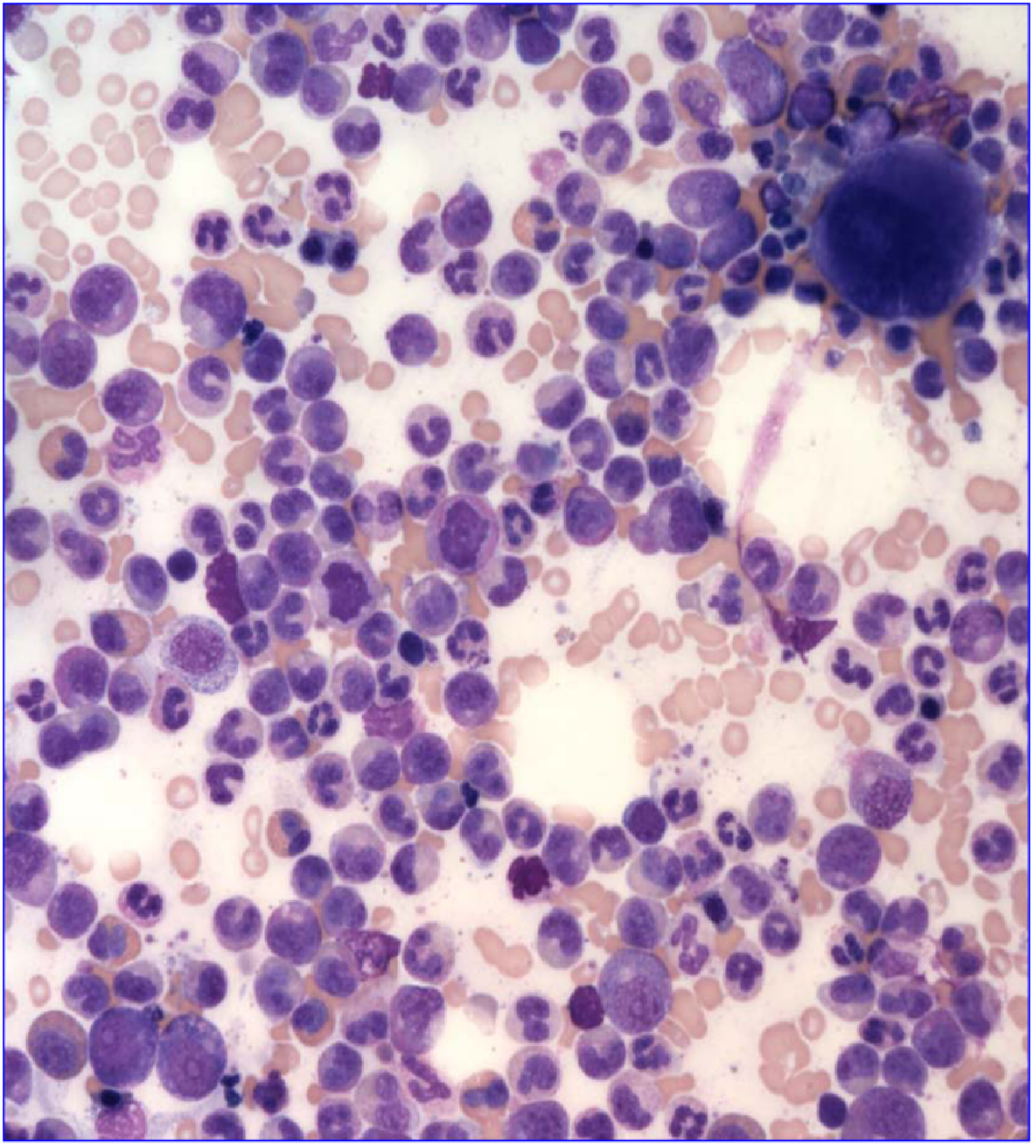
**Bone marrow aspiration analysis showing striking myeloid hyperplasia with complete granulocytic maturation to segmented neutrophils. **Megakaryocytes were adequate in number without overt cytologic atypia although a few hypolobated forms were present. There were no lymphoid infiltrates seen.

**Figure 2:**
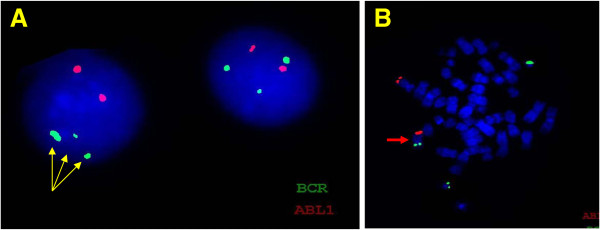
**A BCR-ABL1 FISH for Ph chromosome revealed normal hybridization pattern [negative for t(9,22)(q34;q11.2) BCR/ABL1 fusion]. **However, a third signal for 22q11.2 (*BCR*) probe was observed in 61 % of cells in interphase (panel **A**: green signal; arrows), suggestive of an extra chromosome 22 or additional chromosome material containing the 22q11.2 region (panel **B**; red arrow).

**Figure 3:**
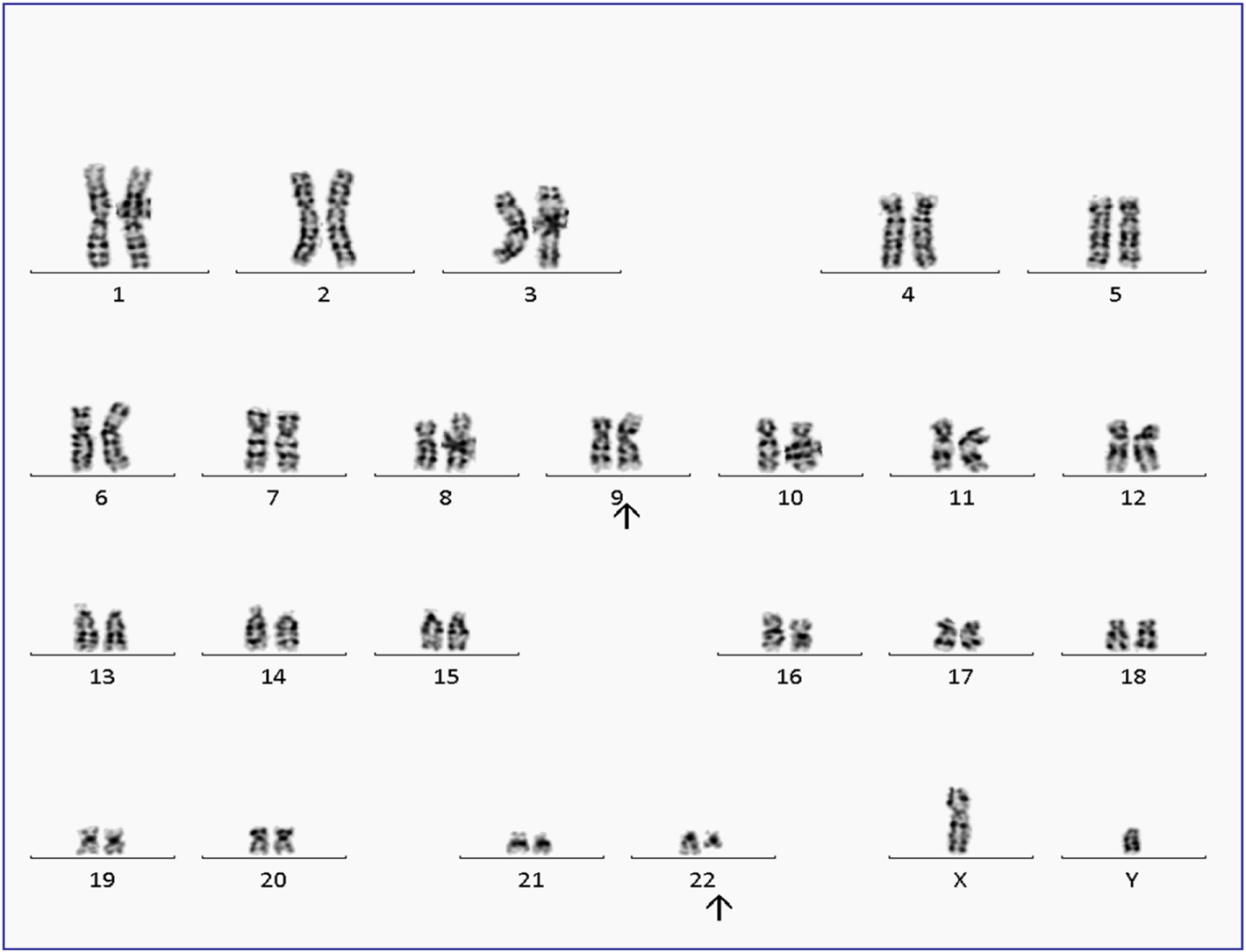
**G-banded chromosome analysis (bone marrow) revealed an abnormal 46,XY, t(9;22)(p24;q11.2)[18]/46,XY [**[2]**] karyotype; an apparently balanced translocation between the short arm of one chromosome 9 and the long arm of one chromosome 22, which was detected in 90 % of metaphases analyzed.**

The patient is an 84 year-old male, who first presented in October 2003 with complaints of fatigue, a 20 pound weight loss over a two month time period, occasional night sweats, leukocytosis (98,6 k/uL with a predominance of neutrophils and less mature myelocytes and metamyelocytes), anemia (Hb 10.9 g/dL), and normal platelets count (283 k/uL). Physical exam was remarkable for a protuberant abdomen with hepatosplenomegaly and bilateral pitting edema at the mid calves. Routine labs showed an elevated white blood cell count of 36,600, low hemoglobin of 10.32 g/dL and normal platelets of 275 k/uL. His differential showed 71.8% neutrophils, 7.2% lymphocytes, 11.6% monocytes, 2.9% eosinophils and 6.5% basophils. Bone marrow aspiration and biopsy showed hypercellularity with striking myeloid hyperplasia with complete granulocytic maturation to segmented neutrophils (Figure 1). Only rare erythroid precursors were present and their maturation was normoblastic without nuclear: cytoplasmic dyssynchrony. Megakaryocytes were adequate in number without overt cytologic atypia and few hypolobated forms present. There were no lymphoid infiltrates seen. Flow cytometry showed hypogranular maturing myeloids with no evidence of an increase in myeloid blasts. Fluorescence in-situ hybridization (FISH) and real time RT-PCR were both negative for BCR/ABL1 fusion gene (Figure 2). Chromosome analysis showed a male chromosome complement with an atypical translocation between the short arm of chromosome 9 and the long arm of chromosome 22 [t(9;22)( p24;q11.2)] (Figure 3).

The patient was started on allopurinol 300 mg daily and hydroxyurea 500 mg twice daily for presumed chronic myelogenous leukemia in the chronic phase. After two weeks of treatment, his white blood cell count decreased to 3,000 with an absolute neutrophil count of 2,320, his hemoglobin decreased to 8 g/dL, and his platelets decreased to 54 k/uL. His hydroxyurea was held for two weeks and on a return visit, his WBC had climbed to 7,000 with an absolute neutrophil count of 5,090, hemoglobin increased to 10.8 g/dL after 2 units of packed red blood cells, and platelets increased to 168 k/uL. The patient was lost to follow up until September 2005 when he was hospitalized for a bleeding gastrointestinal ulcer. His WBC count increased to 22,000 without treatment, but the patient was started on imatinib 400 mg twice daily at that time and was then once again lost to follow up till the current visit.

In June 2010, the patient presented with moderate normocytic normochromic anemia (9.8 g/dL), normal platelet count (332 k/uL), and high total leukocyte count (32.4 k/uL) composed mainly of left-shifted granulocytes. A repeat bone marrow aspiration and biopsy showed hypercellularity and marked myeloid hyperplasia with a mild left shift, mild dyserythropoiesis, and <5% blasts. Megakaryocytes were again adequate in number and morphology with no dysplastic changes. Cytogenetic examination of the patient’s bone marrow aspirate by conventional G-banding analysis was performed on 2 unstimulated short-term cultures (24 and 48 hrs). Chromosome analysis showed the translocation (9;22)(p24;q11.2) as a sole abnormality in 90% (18/20) of analyzed metaphases. To exclude subtle BCR/ABL1 fusion due to three-way translocation or insertion translocation, FISH assay was performed using dual fusion probes (from Abbott Molecular-Vysis, Des Plaines, IL) for 9q34 (*ABL1*) and 22q11.2 (*BCR*) regions and excluded BCR-ABL1 fusion; however an extra signal for the BCR probe was observed in 61% of interphase nuclei. No mutations were seen in *JAK2* exons 12–14 by Sanger sequencing.

## Molecular Analysis

### RT-PCR and Sequencing of BCR-JAK2 Fusion Transcript

A potential BCR-JAK2 fusion was suspected based on the chromosome analysis revealing a translocation t(9;22)( p24;q11.2) and clinical diagnosis of MPD. Total RNA was isolated from patient’s EDTA plasma sample by EasyMag® extraction kit (BioMérieux, Durham, NC) following manufacturer’s instructions. A total of six individual RT-PCR reactions were designed to determine the possible breakpoints within *BCR* and *JAK2* resulting in a fusion transcript. The RT-PCR was performed using SuperScript™ III one step RT-PCR systems with Platinum® *Taq* DNA polymerase (Invitrogen, Carlsbad, CA). The PCR conditions were as follows: initial annealing step at 55°C for 30 min and 94°C for 2 min, followed by 40 cycles of 94°C for 15 second, 60°C for 30 second and 68°C for 1 min and a final extension step of 68°C for 7 min. Specific PCR products, were purified by MinElute gel extraction (MinElute™ Gel Extraction Kit, Qiagen Cat. # 28606). The PCR products were then sequenced in both forward and reverse directions using ABI PRISM® 3730XL genetic analyzer (Applied Biosystems, Foster City, CA). Sequencing data are base-called by Sequencing Analysis software and NCBI blast website (http://www.ncbi.nlm.nih.gov/blast/). RT-PCR was performed using forward primers mapping to the coding sequences of exons 1 (5’-CCT CGC AGA ACT CGC AAC A-3’), 13 (5’-GAG CTG CAG ATG CTG ACC AA-3’), and 19 (5’-TGAAGGCAGCCTTCGACG-3’) of the minor, major, and micro breakpoint regions of the *BCR* locus, respectively [NC_000022.10]. We used 2 reverse primers mapping to exon 15 (5’- CCA TGC CAA CTG TTT AGC AA-3’) and exon 20 of *JAK2* gene (5’- TCA TAC CGG CAC ATC TCC ACA C-3’) [NC_000009.11] [5,6].

## Results

**Figure 4:**
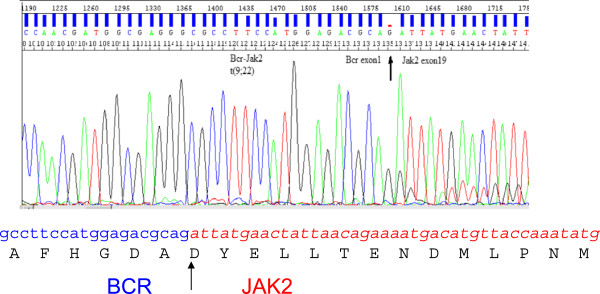
**Direct sequencing of the RT-PCR product and sequence alignment revealed a break at nucleotide 1279 in *BCR *(nucleotides highlighted in blue) and at nucleotide 2434 in *JAK2 *(nucleotides highlighted in red and italicized); the fusion product included the entire exon 1 of *BCR *fused to nt 1 of exon 19 of *JAK2 *in frame.**

A presumptive diagnosis of MPD and possible BCR-JAK2 fusion was suspected from chromosome and FISH analysis revealing a translocation t(9;22)( p24;q11.2). Confirmation and delineation of the fusion was pursued by additional molecular analysis. A specific amplification product of approximately 340 bp was obtained from the RT-PCR reaction. Direct sequencing of the RT-PCR product and sequence alignment revealed a fusion at nucleotide 1279 of *BCR* and at nucleotide 2435 of *JAK2* coding transcripts, respectively; the fusion product included the entire exon 1 of *BCR* fused to nt 1 of exon 19 of *JAK2*, in frame (Figure 4). There was no loss or insertion of a base at the breakpoints. This would predict a break upstream of exon 1 at the *BCR* genomic locus and within intron 18 of *JAK2* locus. The breakpoint within the *BCR* gene corresponds to the minor breakpoint cluster region that results in the p190 BCR-ABL fusion protein in CML [12,13]. The in-frame fusion product is predicted to produce a 747 amino acid protein. The predicted protein product likely includes the coiled–coil oligomerization domain of BCR and the segment immediately distal to the JH2-pseudokinase domain of *JAK2*, thus preserving its active protein tyrosine kinase domain.

## Conclusions

Though relatively rare and likely under-diagnosed, the BCR-JAK2 fusion event in this case with CML/MPD adds to the spectrum of rare yet recurrent translocation partners for each of the genes, respectively. The *BCR* gene harbors two common breakpoints involved in the formation of the two alternative forms of the Philadelphia chromosome translocation seen in chronic myeloid leukemia and acute lymphoblastic leukemia [14]‐[16]. These alternative breakpoints result in fusion of different exon sets of *BCR* to a common subset of the exons of the *ABL1* gene located on chromosome 9 [p210(BCR-ABL) and p190(BCR-ABL)] with constitutive activation of ABL tyrosine kinase.

*JAK2* kinase is a member of a family of non-receptor tyrosine kinases involved in non-catalytic cytokine receptor signaling. The common gain-of-function mutation, V617F, has been strongly associated with polycythemia vera, essential thrombocythemia, and primary myelofibrosis. Rare translocations involving *JAK2* and resulting in fusion transcripts with oncogenic potential have been described in ALL and CML. Interestingly, the Drosophila Janus Kinase homolog, hopscotch (hop) gene, influences proliferation and differentiation of various cell types, particularly in hematopoietic lineages; mutations in the Drosophila hopscotch (hop) gene also cause proliferative defects [17].

These data provide evidence in support of a leukemogenic role for BCR-JAK2 fusion in myeloproliferative disorders, including CML, and complements data provided by the first case report by Griesinger et al. [11]. To our knowledge this represents the second case of CML-like MPD with a translocation resulting in BCR-JAK2 fusion. Interestingly, this case may also suggest the potential recurrent nature of the chromosomal breakpoints and resulting in fusion between *JAK2* and *BCR* genes. Breaks and fusions between the serine/threonine kinase BCR gene and tyrosine kinase *JAK2* result in a fusion gene with a potential for constitutive kinase activity [3]. This is accompanied by disruption of the normal functions of the individual counterparts. Fusion of the oligomerization domain of *BCR* with the crucial tyrosine kinase domain of JAK2 could be predicted to possess significant oncogenic potential. The N-terminal oligomerization domain of BCR is essential for the oncogenicity of the Bcr-Abl protein. Though speculative, it may be reasonable to predict that an intact tyrosine kinase domain of JAK2, under the influence of the BCR oligomerization domain, would lead to phosphorylation and constitutive activity of JAK2 kinase activity and downstream oncogenic effects. Similar speculative predictions have been proposed for oncogenic ETV6-JAK2 fusion [4,10]. The impact of tyrosine kinase inhibitor (TKI) therapy in cases with *JAK2* mutations and translocations is still unclear and likely ineffective in the few cases reported with translocations. However, in this case, Imatinib therapy was initiated during the second encounter (two years post-diagnosis). Loss to follow-up for the following five years precludes any conclusions regarding the effect, or lack thereof, of Imatinib in this patient.

This report, complemented by data from previous cases, strongly suggests shared pathways between *JAK2* activation and oncogenic events resulting in ALL, CML and probably additional lympho- and myeloproliferative disorders. This makes it imperative to utilize multiple diagnostic tools (chromosomes, FISH, etc.,) to adequately investigate hematologic malignancies. Identification of additional cases will provide the opportunity to draw more explicit genotype-phenotype correlations and implement beneficial therapeutic regimens.

## Consent to publish

Written informed consent was obtained from the patient for publication of this Case report.

## Competing interests

The author(s) declare that they have no competing interests.

## Authors’ contributions

MME, RR, SA, IZ and AL contributed to conception and design, acquisition of data, analysis and interpretation of data. RR, IZ, AL were involved in the clinical evaluation, management and long-term follow-up. MME, TS, AG, WM and SA were responsible for the pathological, cytogenetic and molecular analysis of data and results. MME and TS were involved in the manuscript preparation and finalization. All authors read and approved the final manuscript.
